# Machine Learning Framework for Conotoxin Class and Molecular Target Prediction

**DOI:** 10.3390/toxins16110475

**Published:** 2024-11-03

**Authors:** Duc P. Truong, Lyman K. Monroe, Robert F. Williams, Hau B. Nguyen

**Affiliations:** 1 Theoretical Division, Los Alamos National Laboratory, Los Alamos, NM 87545, USA; 2 Bioscience Division, Los Alamos National Laboratory, Los Alamos, NM 87545, USA

**Keywords:** conotoxins, machine learning, collisional cross section, post-translational modifications, prediction, receptors, ion channels, conotoxin class

## Abstract

Conotoxins are small and highly potent neurotoxic peptides derived from the venom of marine cone snails which have captured the interest of the scientific community due to their pharmacological potential. These toxins display significant sequence and structure diversity, which results in a wide range of specificities for several different ion channels and receptors. Despite the recognized importance of these compounds, our ability to determine their binding targets and toxicities remains a significant challenge. Predicting the target receptors of conotoxins, based solely on their amino acid sequence, remains a challenge due to the intricate relationships between structure, function, target specificity, and the significant conformational heterogeneity observed in conotoxins with the same primary sequence. We have previously demonstrated that the inclusion of post-translational modifications, collisional cross sections values, and other structural features, when added to the standard primary sequence features, improves the prediction accuracy of conotoxins against non-toxic and other toxic peptides across varied datasets and several different commonly used machine learning classifiers. Here, we present the effects of these features on conotoxin class and molecular target predictions, in particular, predicting conotoxins that bind to nicotinic acetylcholine receptors (nAChRs). We also demonstrate the use of the Synthetic Minority Oversampling Technique (SMOTE)-Tomek in balancing the datasets while simultaneously making the different classes more distinct by reducing the number of ambiguous samples which nearly overlap between the classes. In predicting the alpha, mu, and omega conotoxin classes, the SMOTE-Tomek PCA PLR model, using the combination of the SS and P feature sets establishes the best performance with an overall accuracy (OA) of 95.95%, with an average accuracy (AA) of 93.04%, and an f1 score of 0.959. Using this model, we obtained sensitivities of 98.98%, 89.66%, and 90.48% when predicting alpha, mu, and omega conotoxin classes, respectively. Similarly, in predicting conotoxins that bind to nAChRs, the SMOTE-Tomek PCA SVM model, which used the collisional cross sections (CCSs) and the P feature sets, demonstrated the highest performance with 91.3% OA, 91.32% AA, and an f1 score of 0.9131. The sensitivity when predicting conotoxins that bind to nAChRs is 91.46% with a 91.18% sensitivity when predicting conotoxins that do not bind to nAChRs.

## 1. Introduction

Conotoxins are small and highly potent neurotoxic peptides derived from the venom of marine cone snails which have captured the interest of the scientific community due to their pharmacological potential [1,2]. These toxins display significant sequence and structure diversity, which results in a wide range of specificities for several different ion channels and receptors [3]. As a result of their specific binding to ion channels, some conotoxins have already been developed into therapeutic agents, such as the pain reliever ziconotide [4].

Despite the recognized importance of these compounds, our ability to determine their binding targets and toxicities remains a significant challenge. To effectively characterize conotoxins, it is necessary to employ a variety of intricate and demanding experimental techniques [5]. When the difficulty of experimental characterization is coupled to the thousands of new peptide sequences obtained through transcriptomics and proteomics, a significant bottleneck arises in the identification and prediction of conotoxin molecular targets and protentional therapeutic applications.

Given the demand for high-throughput methods for the characterization of conotoxins, a natural approach is to apply computational techniques to accelerate the process. Direct toxicity prediction of conotoxins from sequence, and even sequence and structure in many cases, remains out of reach for current computational methods [6]. Two critical steps for toxicity prediction are the sorting of conotoxins into classes and the determination of their target receptors. Successful methods to accomplish these steps pave the way to solving toxin–target complex structures as well as finally predicting receptor binding affinities and compound toxicity.

Predicting the molecular targets of conotoxins, based solely on their amino acid sequence, remains a formidable challenge due to the intricate relationships between structure, function, target specificity, and the significant conformational heterogeneity observed in conotoxins with the same primary sequence such as AuIB [7], BuIA [8,9], and GI [10]. Nevertheless, function prediction is critical to harness the therapeutic potential of these molecules and streamlining the discovery of new conotoxin-based drugs. Traditional sequence alignment and motif-based methods provide some insights into conotoxin–receptor interactions [11]; however, the inclusion of dipeptide compositions, which encapsulates local sequence information of the peptide, is necessary to achieve successful predictive methods [12,13].

The advent of machine learning (ML) in bioinformatics has revolutionized predictive modeling for a wide variety of molecular functions [14], and conotoxins have been no exception to this trend [6,15]. Recently, a variety of ML-based methods have been proposed to predict the functions of conotoxins. For instance, Yuan, et al. [12] used support vector machines (SVMs) to predict the ion channel targets of conotoxins, implementing sequence-derived features including amino acid compositions and dipeptide compositions. Xianfang et al. [13] underscored the importance of dipeptide composition in predicting conotoxin functions by demonstrating that integrating dipeptide data with sequence information provides a deeper, more precise representation of peptide structure and conformation. These studies collectively demonstrate how ML can tap into the predictive potential hidden in the intricate data structures and conformations of peptide sequences.

**Figure 1:**
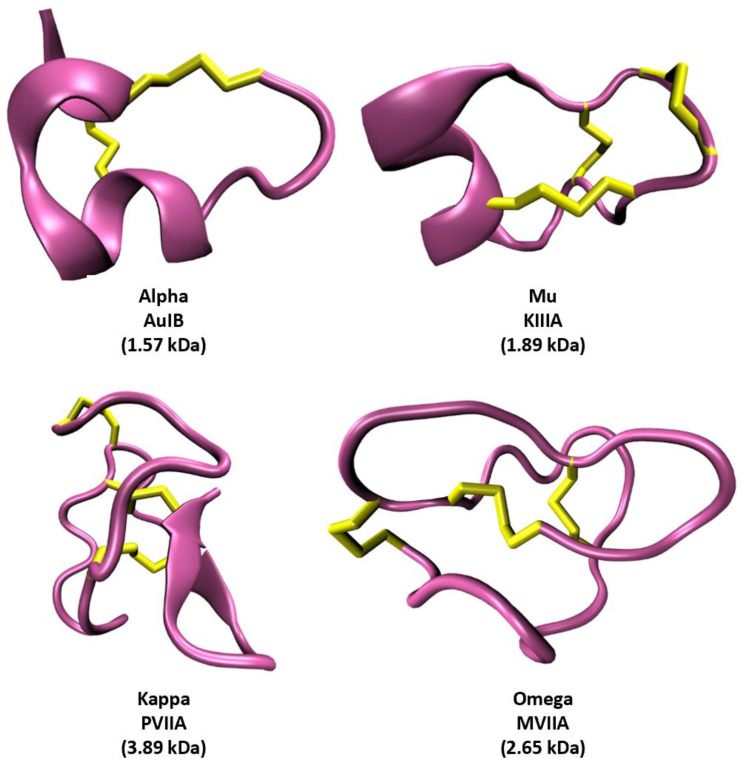
Example structures of the alpha, mu, kappa, and omega classes (pharmacological families) of conotoxins. The backbone structures are shown in a pink cartoon representation. Disulfide bridges are shown in yellow. Class, toxin name, and mass are given below each structure. PDB references are 1MXN [7], 7SAV [16], 1DW4 [17], and 1AV3 [18] clockwise from top left.

**Figure 2:**
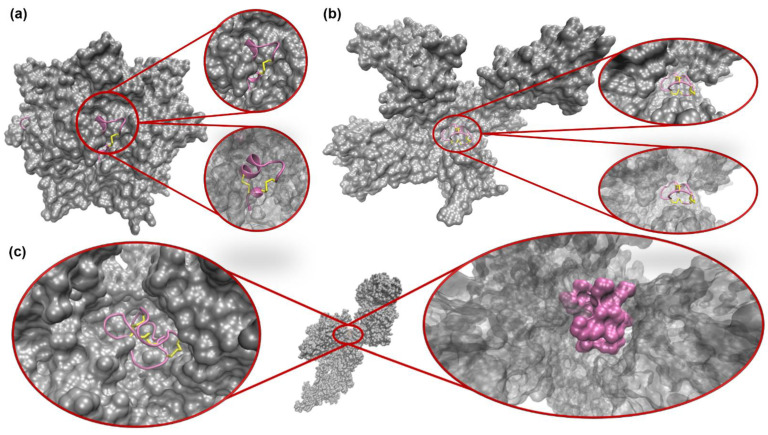
Samples of different conotoxin classes bound to their target receptors. (**a**) Alpha conotoxin PNIA (PDB: 2BR8 [19]) bound to the Acetylcholine binding protein (AChBP). To the left, a complex structure shows the toxin in pink, its disulfide bonds in yellow, and the AChBP in silver. To the right, circles are zoomed-in to show the same binding site, but the bottom circle shows a transparent receptor to more easily see the conotoxin conformation. (**b**) Mu conotoxin KIIIA (PDB: 6J8E [20]) bound to the voltage gated sodium channel Nav1.2-beta2, with the right showing similar zoomed in perspectives as (**a**). (**c**) Omega conotoxin MVIIA (PDB: 7MIX [21]), marketed as ziconotide, is shown in its complex with the voltage gated calcium channel Cav2.2. The center structure is the conotoxin/ion channel complex with a zoomed-in view of the bound toxin displayed in ribbon representation (**left**) and a zoomed-in view showing the receptor (transparent) and the conotoxin in a surface representation to illustrate the tight, key-like fit of the toxin binding site (**right**).

Though previous work has classified conotoxins by superfamily and predicted conotoxins that target ion channels [15], conotoxin classes (pharmacological families), which display striking conformational variations with differing numbers of disulfide bridges (Figure 1), have not been classified successfully. The heterogeneity observed in the sequences and the conformational structures of conotoxins results in diverse binding modes across distinct ion channels (Figure 2). The diversity and specificity of binding for these toxins make them potential new therapeutics but increases the challenge of characterizing them.

We have previously demonstrated that the inclusion of post-translational modifications (PTMs), collisional cross section (CCS) values, and other structural features, when added to the standard primary sequence features, improves the prediction accuracy of conotoxins against non-toxic and other toxic peptides across varied datasets and several different commonly used ML classifiers [6]. Here, we present the effects of these features on conotoxin class and target receptor predictions, in particular, predicting conotoxins that bind to nicotinic acetylcholine receptors (nAChRs). Due to the small and unbalanced datasets available for this study, we also demonstrate a new ML framework that employs the Synthetic Minority Oversampling Technique (SMOTE) [22] together with Tomek method [23] to more accurately predict conotoxin classes and conotoxins that target nAChRs. SMOTE-Tomek was selected over other sampling methods because it effectively addresses both dataset imbalance and any noise in the data [22], thereby the improving performance of the model [23,24].

## 2. Results

### 2.1. Construction of Datasets

One common challenge with building ML models for biological samples is that the training datasets are usually small because of the sparse experimental biological data that are currently available. This is the case for our datasets, given that only conotoxins with experimentally solved 3-D structures are included. Our initial dataset of conotoxins with solved structures was constructed from entries in the Protein Data Bank [25] (PDB) and the Biological Magnetic Resonance Bank [26] (BMRB). These entries were then grouped into the three most common classes of conotoxins, alpha, mu, and omega. With the delta and kappa classes having only six and four entries, respectively, the algorithm has insufficient data to create meaningful and diverse synthetic points, risking the generation of artificial samples that poorly represent the underlying distribution. The delta and kappa class entries were therefore discarded and not used for training. The distribution of conotoxins in each class and conotoxins that bind or do not bind to nAChRs is shown in Table 1 and Figure S1.

### 2.2. Feature Extraction and Selection

Features were extracted from PDB files using a combination of python and perl scripts as well as obtained from the Define Secondary Structure of Proteins (DSSP) [27,28] and the High Performance Collision Cross Section (HPCCS) [29] programs as previously described [6]. Extracted features were divided into four feature sets (P, P2, SS, and CCS). The P feature set contains 15 sequence-related features that include the frequency of amino acid occurrence and the physiochemical characteristics of all amino acids established by the number of charged, polar, hydrophobic, small, large, aromatic residues as well as by total charge, mass, dipeptide 0 gap, and dipeptide 1 gap. Dipeptide 0 and dipeptide 1 are the frequencies of co-occurring residues in the sequence as adjacent neighbors or with one residue separating them, respectively. Thus, each dipeptide feature contains 400 features, bringing the total number of features in the P feature set to 813. Most of the current ML models use only the P feature set to train ML algorithms [15]. The P2 feature set includes the number of PTMs and frequency of dipeptide 2 gap which are the frequency of residues appearing as neighbors with two residues separating them. By including the PTM amino acids, the dipeptide 2 gap contains 528 features, bringing the total number of features in the P2 feature set to 529. The SS feature set has a total of 16 features consisting of structural data that include the number of residues in any helical secondary structure conformations as defined by DSSP. The CCS feature set consists of 1 feature for each peptide and is calculated by HPCCS program. All features were combined through concatenation to prevent bias towards a specific feature or feature set. Feature concatenation has been widely used to preserve and present all the information from the various features to an ML pipeline, ensuring a comprehensive representation of the data [30]. A complete list of all features in each set is shown in Table S1.

### 2.3. Conotoxin Class Prediction

In order to determine how PTMs, CCS, and structural feature sets affect the classification performance for predicting the alpha, mu, and omega conotoxin classes, these feature sets were tested either individually or in combination with other feature sets, using four different ML classifiers: Penalized Logistic Regression (PLR) [31], SVM [32], Random Forest (RF) [33], and xGBoost (xGB) [34]. Due to the highly unbalanced datasets for these three conotoxin classes used in this study, the oversampling technique, SMOTE [22], was used to balance the conotoxin class distributions by generating artificial data for the minority classes. SMOTE works by generating synthetic samples along the line segments that connect minority class samples, which fills in the gaps between minority class samples, and densifies the minority clusters. In this way, noisy samples from the minority class are added to the data, which increases the sample size without duplicating the samples in the classes. This helps to increase the representation of the minority mu and omega conotoxin classes, making them more comparable to the majority alpha conotoxin class. However, to avoid overfitting the ML models, due to overlapping samples between each of the classes, SMOTE-Tomek was used together as a hybrid method, combining both undersampling and oversampling techniques to clean up overlapping samples. Tomek links are pairs of instances, one from the majority class and one from the minority class, that are similar to each other but belong to the different classes [24]. These pairs can be considered as noisy or borderline examples. Tomek links can be removed from the dataset to improve the separation between the classes; however, by combining SMOTE and Tomek techniques, a more balanced and representative dataset was created, leading to better classification performance in our imbalanced dataset scenarios. The sample sizes for the three classes of conotoxins were similar after SMOTE-Tomek was applied to the datasets, indicating that a more balanced dataset was constructed (Table S2).

**Figure 3:**
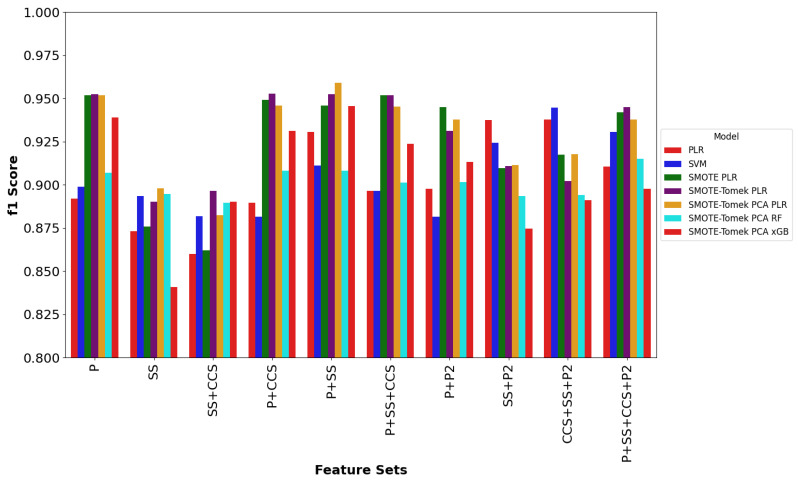
Comparison plots of f1 scores obtained from different ML models for the different feature sets and feature set combinations in predicting alpha, mu, and omega conotoxin classes using different ML models.

In addition, due to the small dataset sizes, models were tested using leave-one-out cross validation in which the models were trained using all but one entry and then tested with the entry that was left out [15,35]. This method helps to reduce the variability in the F-score by averaging results across all possible splits, leading to a more stable and reliable performance estimate. The cross validation was then repeated leaving a different entry out each time [15,35]. Four different classifiers: SVM, PLR, RF, and xGB, were coupled with different procedures to create various models to predict the three conotoxin classes using our different feature sets. The f1 scores, as shown in Table 2 and Figure 3, obtained for each model were used to evaluate the classification performance as detailed in the Section 4. Higher f1 values indicate better prediction performance.

The results show that the SS feature set alone, or in combination with the CCS feature set (SS + CCS) did not increase the prediction performance, compared to the P feature set alone. Similarly, the addition of the CCS feature set on top of the P feature set (P + CCS), the addition of the P2 feature set on top of the P feature set (P + P2), or the addition of CCS and SS feature sets on top of the P feature set (P + SS + CCS) did not significantly affect the performance of all models tested. Interestingly, the addition of the SS feature set on top of the P2 feature set (SS + P2), the addition of CCS and SS feature sets on top of the P2 feature set (CCS + SS + P2), or the addition of CCS, SS, and P2 feature sets on top of the P feature set (P + SS + CCS + P2) increased the F-score for both the PLR and SVM models, but decreased the performance of the other five models. This was true except for the (P + SS + CCS + P2) feature set using the SMOTE-Tomek PCA RF model, which showed an f1 increase of 0.0078, compared to using the P feature set alone. Notably, adding the SS feature set on top of the P feature set (P + SS) increased f1 scores across all seven models tested (except for the SMOTE-PLR model) and the best f1 score of 0.9590 was obtained with the SMOTE-Tomek PCA PLR model. Moreover, the SMOTE-Tomek PLR model outperformed the SMOTE-PLR when single feature sets were used or when they were combined with each other, indicating that the SMOTE-Tomek hybrid technique improved the overall performance of the models tested.

As shown in Table 2, the SMOTE-Tomek PCA PLR model, overall, has the best performance in predicting conotoxin classes, across multiple different feature sets and feature set combinations. This model, therefore, was used to classify the alpha, mu, and omega conotoxin classes. Metrics, including overall accuracy (OA), average accuracy (AA), sensitivity (Sn), and f1 score for each class were used to evaluate the classification performance as indicated in the Section 4. Higher values for these metrics indicate better performance.

Table 3 shows the effect of different feature sets and feature set combinations on the performance of the SMOTE-Tomek PCA PLR model to predict the three conotoxin classes. These results demonstrate that the SS features alone, or in combination with the CCS feature set (SS + CCS) did not improve prediction accuracy, compared to the P feature set alone. Similarly, the addition of the CCS feature set on top of the P feature set (P + CCS), the addition of P2 feature set on top of the P feature set (P + P2), or the addition of CCS and SS feature sets on top of the P feature set (P+ SS + CCS) also did not have any effect on prediction accuracy. The addition of the P2 feature set did not increase prediction accuracy. Specifically, when the P2 feature set was combined with the SS feature set (SS + P2), with both the SS and CCS feature sets (CCS + SS + P2), or when all feature sets were combined (P + SS + CCS + P2), none of the four metric scores (OA, AA, Sn and f1) were improved compared to just using the P feature set alone. However, when the SS feature set was added on top of the P feature set (P + SS), the OA was increased by 0.68%, the AA by 1.15%, and the f1 score by 0.007, obtaining the same sensitivity for the alpha and omega classes as when using the P feature set alone. The sensitivity for predicting the mu conotoxin class was also increased by 3.45%. However, the sensitivity for predicting the omega conotoxin class was the same regardless of the feature sets and feature set combinations used.

The best average accuracy for SMOTE-Tomek PCA PLR model, 93.04%, was also achieved when using the combination of P and SS feature sets (P + SS). In addition, the SS feature set improved the prediction sensitivity for the mu conotoxin class, suggesting that there might be some distinct information in the SS feature set that may help to distinguish the mu conotoxin class further.

### 2.4. Prediction of Conotoxins That Target nAChRs

Next, the feature sets were evaluated for their effect on the classification performance of conotoxins that bind to nAChRs. Similar to the approach in Section 2.3, to determine the best model for conotoxin class prediction, several different models were tested; however, since the performance of the models using the hybrid SMOTE-Tomek technique considerably improved the conotoxin class prediction, only models using this protocol are presented here. The f1 scores, obtained for each feature set, and the feature set combinations for each model used in predicting conotoxins that bind to nAChRs are shown in Table 4.

As shown in Table 4, when the P feature or the SS feature sets were used by themselves, added on top of the P2 feature set (P2 + SS), or in combination with the CCS feature set (SS + CCS), the prediction performance was not improved across the five models tested, except for the SMOTE-Tomek PCA RF model. Similarly, the addition of CCS and SS feature sets on top of the P2 feature set (CCS + SS + P2) or the combination of all feature sets (P + SS + CCS + P2), except for the SMOTE-Tomek PCA RF model, also did not improve the performance of the other models tested.

Interestingly, the addition of the SS feature set (P + SS), or the addition of the CCS and SS feature sets (P + SS + CCS) increased the performance of both the SMOTE-Tomek PCA RF and the SMOTE-Tomek PCA SVM models but did not increase the performance of the other four models. Adding the P2 feature set on top of the P feature set (P + P2) increased the performance of only half of the models tested. The addition of the CCS feature set on top of the P feature set (P + CCS) improved the performance of two of the six models tested. The f1 score (0.9131) for the SMOTE-Tomek PCA SVM model with the (P + CCS) feature set was the highest score obtained for any of the feature sets and feature set combinations, across all six models tested. Consequently, the SMOTE-Tomek PCA SVM model was chosen for further evaluation in predicting conotoxins that bind to nAChRs since it showed the highest performance across multiple feature set combinations (Table 4).

The SMOTE-Tomek PCA SVM model was evaluated for its ability to classify the conotoxins that bind to nAChRs based on the OA, AA, Sn, and f1 score as indicated in the Section 4. The effect of different feature sets and feature set combinations on the performance of the SMOTE-Tomek PCA SVM model is shown in Table 5. The results demonstrate that the SS feature alone, or in combination with the CCS feature set (SS + CCS) did not improve the model performance, across all four metrics when compared to the P feature set alone. Similarly, the addition of the P2 feature set on top of the P feature set (P + P2), the addition of the SS feature set on top of the P2 feature set (P2 + SS), the addition of CCS and SS feature sets on top of the P2 feature set (CCS + SS + P2) or when all feature sets are combined (P + SS + CCS + P2) did not improve the model performance, based on all four metric values. However, these feature set combinations showed an increase in sensitivity of 1–2% when predicting conotoxins that do not bind to nAChRs.

Notably, when compared to just the P feature set, the addition of the CCS feature set (P + CCS), the addition of the SS feature set (P + SS), or the addition of both the SS and CCS feature sets (P + SS + CCS), increased all three metric scores OA, AA and f1, in addition to 1–2% increase in sensitivity when predicting conotoxins that do not bind to nAChRs. The (P + CCS) feature set combination had the highest overall performance metrics, with an AA score of 91.32% (an increase of 1.71%), a highest OA score of 91.30% (an increase of 1.63%), and an f1 score of 0.9131, (an increase of 0.0163), when compared to just the P feature set alone. In addition, the use of (P + CCS) feature set combination also increased the sensitivity in predicting conotoxins that bind to nAChRs by 2.46% and in predicting conotoxins that do not bind to nAChRs by 0.98%.

## 3. Discussion

We have demonstrated that the implementation of the hybrid SMOTE-Tomek technique in all models improved their performance in predicting conotoxin classes and conotoxins that bind to nAChRs. In predicting the alpha, mu, and omega conotoxin classes, the SMOTE-Tomek PCA PLR model, using the combination of the SS and P feature sets establishes the best performance with an OA of 95.95%, an AA of 93.04%, and an f1 score of 0.959. Using this model, we obtained sensitivities of 98.98%, 89.66%, and 90.48% when predicting alpha, mu, and omega conotoxin classes, respectively. Similarly, in predicting conotoxins that bind to nAChRs, the SMOTE-Tomek PCA SVM model, which used the CCS and the P feature sets, demonstrated the highest performance with a 91.3% OA, a 91.32% AA and an f1 score of 0.9131. The sensitivity when predicting conotoxins that bind to nAChRs is 91.46% with a 91.18% sensitivity when predicting conotoxins that do not bind to nAChRs. The effectiveness of the hybrid SMOTE-Tomek technique, when applied to conotoxin class prediction, is not surprising given the challenges of working with conotoxin data sets that are data sparse, leading to imbalanced training datasets. The use of the SMOTE-Tomek aids in balancing the datasets while simultaneously making the different classes more distinct by reducing the number of ambiguous samples which nearly overlap between the classes.

Interestingly, when predicting the omega conotoxin class, the sensitivity is the same (90.48%) across different feature sets and feature set combinations. Examining these results, we found that the model consistently misclassified two entries in the omega class. One is the conotoxin Eb1.6, which resembles an alpha conotoxin but inhibits the N-type calcium ion channel as shown in the BMRB [26] and the other omega conotoxin is MVIIA (pdb code: 1dw5). Perhaps, slightly different structural conformations in MVIIA conotoxin and the difference in amino acid sequence of Eb1.6 from the rest of the entries are the main causes leading to these misclassifications. Further investigation, including removing conotoxin Eb1.6 and MVIIA entries one at a time from the omega class dataset should be carried out to identify their effect on ML model performance.

We have also shown that the addition of the SS and CCS feature sets, on top of the P feature set, increased prediction accuracy. In particular, compared to the use of the P feature set alone, the addition of the SS feature set was found to have the highest performance in predicting the three alpha, mu, and omega conotoxin classes. Similarly, the addition of the CCS feature set, on top of the P feature set, had the highest performance in predicting conotoxins that bind to nAChRs. The P feature set is expected to be of high importance since the peptides from the same class may naturally share a high sequence similarity with only slight mutations among the population to conserve function within the evolutionary trajectory. The SS feature set is essential in dictating the structural features of the peptides, and as a result, has a high impact in discriminating conotoxins between classes. The peptide sequences dictate sidechain interaction compatibility when they interact with their respective targets, while the CCS feature, a function of shape, size, and charge, distinguishes between toxins according to what fits into the receptor binding pocket, and thus plays an important role in predicting the target receptors, in this case, for nAChRs. Conotoxins are short amino acid sequences but extremely diversified in both three dimensional structures and, consequently, the receptors and ion channels that they target. The number of disulfide bonds and specific disulfide bond patterns define their distinct structures and conformations dictating the types of molecular targets that are representative of each class of conotoxins. The improvement of the prediction accuracy upon the addition of the SS and CCS feature sets, therefore, has strong implications on the biological significance of the conotoxins as these features represent specific conotoxin structures, shapes, sizes and charges, thus enhancing the structure and function relationship in our models.

The increased accuracy observed using the SMOTE-Tomek models, together with the added effect of new feature sets, has exciting implications for peptide-based drug discovery. Conotoxins possess a rich potential for therapeutic applications, given their diverse pharmacological profiles, and the ability to rapidly and accurately screen conotoxins for their class and receptor target is revolutionary allowing identification of potential therapeutic leads. Our results also suggest that there are conserved chemical and structural signatures across conotoxins that distinguish them into different classes that target different host receptors. The acquisition of new, additional experimental data on conotoxin structures and functions is necessary to expand training datasets and to increase the impact of CCS and other structural features. Although the ML framework presented here improves accuracy when predicting conotoxin classes and conotoxins targeting nAChRs, future experimental validation must be randomly carried out to verify the accuracy of the predicted ML models. Such experiments might include both structural and functional characterization to determine three-dimensional structures that target receptors and possible toxicities for unknown conotoxins. While our models were trained to predict the three classes of alpha, mu, and omega conotoxins, in practical applications, a confidence threshold could be determined to allow prediction of unknown samples belonging to any one of the three classes the models were trained on or to the other classes of conotoxins.

## 4. Materials and Methods

### 4.1. Construction of Datasets

Structures were collected from the PDB and BMRB as described previously [6], and were further sorted into classes based on the original authors’ classifications. Conotoxins were also sorted into categories of nAChR binders and non-binders based on their descriptions in UniProt [36].

### 4.2. Feature Extraction

Features were extracted to capture sequence and structural information using perl and python scripts, as well as the DSSP and HPCCS software (version 1.0). Features include amino acid frequency, amino acid type frequencies, secondary structure content, physiochemical surface characteristics, radius of gyration, and CCS values.

### 4.3. Dimensionality Reduction Procedures

Dimensionality reduction procedures are applied to high dimensional data [37], i.e., when the number of features is higher than the number of samples. This is especially useful for our small conotoxin datasets. We have exploited the following dimensionality reduction procedures.

#### 4.3.1. F-Score

F-score is a metric that measures the classifying power of features, given the label of the samples. For each feature, the F-score is defined as ratio of variance between classes over variance within classes. A larger F-score indicates a higher classifying power. However, F-score is a univariate feature selection algorithm, which cannot measure the classifying power of a group of features.

#### 4.3.2. Redundant Feature Elimination

One effective preprocessing step is to remove highly correlated features [38]. This helps other feature selection algorithms to avoid selecting only a group of highly similar features and results in the creation of a dataset with diverse information. To help identify redundant features, Pearson correlation coefficients are computed between all features. If the correlation between two features is larger than a threshold, the feature with the smaller F-score is removed. This procedure produces a smaller dataset, but with an independent set of features. This preprocessing step is similar to Analysis of Variance Correlation (AVC) described by Xianfang et al. [13].

#### 4.3.3. Principle Component Analysis

Principal Component Analysis (PCA) is a widely used dimensionality reduction technique and data analysis method in the field of statistics and ML [22]. Its primary objective is to simplify complex data by transforming it into a new coordinate system where the variance of the data along each axis is maximized. This process allows for the identification of the most significant patterns, structures, or features within the data, making them easier to visualize, analyze, and interpret.

#### 4.3.4. Regularization

Regularization is not a dimensionality reduction method, per se, but is often used to limit the complexity of models. Here, we also couple regularization with some classifiers to create a lower complexity model, which is suitable for our small dataset.

### 4.4. Classifiers

We used four primary classifiers: PLR [31], SVM [32], RF [33], and GB [34]. These classifiers were coupled with other procedures to create the various models.

### 4.5. SMOTE-Tomek

**Figure 4:**
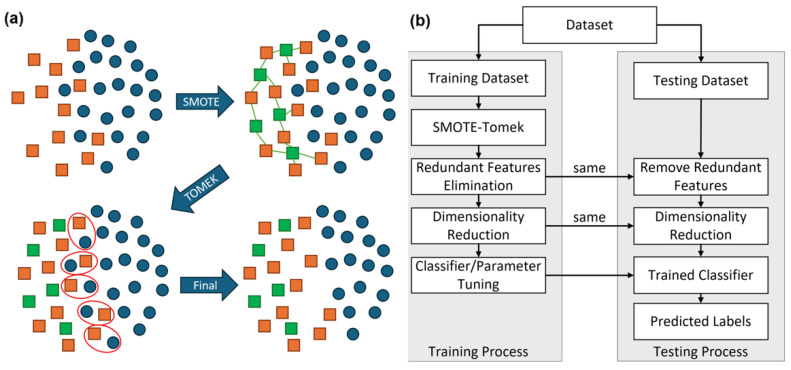
(**a**) A cartoon representation showing how SMOTE-Tomek works together to handle imbalanced datasets. Top left, a mixture of classes, orange squares, and blue circles. The orange squares are underrepresented relative to the circles. Top right, SMOTE produces additional square entries, shown in green, by interpolating between the existing data. Bottom left, Tomek determines pairs for square and circle data (red circle) that are at the boundary between the circle and square classes. Bottom right, entries belonging to the more represented class in the Tomek pairs are removed, and a more evenly balanced and clearly separated training set has been produced. (**b**) Overall ML pipeline describing the process of using a dataset to train and cross validate a classifier.

The SMOTE-Tomek method is a powerful technique in the field of ML and data preprocessing and is specifically designed for addressing the issue of class imbalance in datasets. Class imbalance occurs when one class (the minority class) has significantly fewer samples than another class (the majority class), which can lead to biased models that perform poorly in predicting the minority class. The SMOTE-Tomek method combines two techniques: SMOTE and Tomek links. Tomek links are pairs of instances, one from the majority class and one from the minority class, that are very close to each other but belong to different classes [24]. These pairs can be considered noisy or borderline examples. Tomek links can be removed from the dataset to improve the separation between the classes. By combining oversampling and undersampling techniques, SMOTE-Tomek aims to create a more balanced and representative dataset, leading to better model performance in class-imbalanced scenarios. A cartoon representation of how SMOTE-Tomek work together is illustrated in Figure 4a.

Similar to other data balancing techniques, SMOTE-Tomek was applied only to the training dataset, not the entire dataset, to avoid introducing synthetic data into the test datasets, which should remain untouched to provide an accurate and unbiased evaluation of the models’ performance. As shown in Figure 4b, the dataset was initially split into a training set and a test sample set for the ML pipeline with leave-one-out cross validation. SMOTE was first used to generate synthetic samples for the minority class, balancing the training data. Afterward, Tomek links were applied to remove noisy or borderline samples, particularly those that overlap between classes. The processed dataset was then moved to the next step of the training process, which involved redundant feature elimination.

### 4.6. Performance Evaluation

To ensure consistency across datasets, a leave-one-out (or jack-knife) cross-validation approach to evaluate classifier performance was employed [15]. We assessed classification performance using four metrics: OA, AA, Sn, and f1 score, which are defined as follows:

$$OA=\frac{TP_{0}+TP_{1}}{sample\;size}$$

$$Sn_{i}=\frac{TP_{i}}{TP_{i}+FN_{i}}$$

$$AA=\sum Sn_{i}/number\;of\;classes$$

where $TP_{i}$ and $FN_{i}$ are true positives and false positives for the $i^{th}$ class.

The f1 score is a comprehensive metric to assess the predictive power of a model. It is first defined in the context of one-vs-all:

$$Precision=\frac{TP_{i}}{TP_{i}+FP_{i}}$$

$$Recall=\frac{TP_{i}}{TP_{i}+FN_{i}}$$

$$f1_{i}=2PrecisionRecall/\left(Precision+Recall\right)$$

Then the f1 score for multiclass classification is defined as $f1=\sum w_{i}f1_{i}$ where $w_{i}$ are sample size proportion.

### 4.7. Machine Learning Pipeline

Figure 4 provides an overview of our comprehensive ML pipeline, showing how we utilized the dataset for classifier training and cross-validation. The pipeline consists of four primary stages: over/under-sampling, feature selection, classifier training, and prediction on testing data. To ensure consistency, the jack-knife cross-validation method across all classification tasks was employed.

The only parameter of the classifiers that was fine-tuned was the regularization parameter, and this adjustment was automatically determined during the cross-validation phase within the training process. Subsequently, the trained classifier was applied to the testing samples to make predictions about their labels. To assess performance, we utilized metrics such as OA, AA, Sen, and the f1 score.

## Figures and Tables

**Table 1:** Number of samples in each conotoxin class and number of conotoxins that either bind or do not bind to nAChRs that were used in this study.

Datasets	Sample Sizes
alpha/mu/omega	98 alpha/29 mu/21 omega
nAChRs/non-nAChRs	102 nAChR binders */82 non-nAChR binders

* Note: there are more entries for nAChR binders than for alpha class since some conotoxins from delta, lambda and psi classes also target to nAChRs.

**Table 2:** The f1 scores obtained for different feature sets and feature set combinations on the performance of different models in predicting the alpha, mu, and omega conotoxin classes using different ML models.

	F-Score PLR	F-Score SVM	SMOTE PLR	SMOTE-Tomek PLR	SMOTE-Tomek PCA PLR	SMOTE-Tomek PCA RF	SMOTE-Tomek PCA xGB
P	0.8920	0.8988	0.9520	0.9524	**0.9520**	0.9071	0.9391
SS	0.8732	0.8934	0.8757	0.8902	**0.8981**	0.8948	0.8407
SS + CCS	0.8598	0.8818	0.8621	0.8964	**0.8823**	0.8895	0.8902
P + CCS	0.8897	0.8814	0.9492	0.9528	**0.9459**	0.9083	0.9311
**P + SS**	**0.9307**	**0.9112**	** 0.9459 **	**0.9524**	**0.9590**	**0.9083**	**0.9455**
P + SS + CCS	0.8965	0.8965	0.9519	0.9520	**0.9453**	0.9013	0.9237
P + P2	0.8976	0.8816	0.9449	0.9311	**0.9377**	0.9016	0.9131
SS + P2	0.9376	0.9244	0.9098	0.9100	**0.9116**	0.8935	0.8746
CCS + SS + P2	0.9379	0.9447	0.9173	0.9022	**0.9177**	0.8941	0.8912
P + SS + CCS + P2	0.9107	0.9306	0.9421	0.9449	**0.9377**	0.9149	0.8976

**Table 3:** The effect of different feature sets and feature set combinations on the performance of SMOTE-Tomek PCA PLR model in predicting the alpha, mu, and omega conotoxin classes.

	OA	AA	Sn-Alpha	Sn-Mu	Sn-Omega	f1
P	0.9527	0.9189	0.9898	0.8621	0.9048	0.9520
SS	0.8986	0.8674	0.9388	0.7586	0.9048	0.8981
SS + CCS	0.8851	0.8363	0.9490	0.6552	0.9048	0.8823
P + CCS	0.9459	0.9236	0.9694	0.8966	0.9048	0.9459
**P + SS**	**0.9595**	**0.9304**	**0.9898**	**0.8966**	**0.9048**	**0.9590**
P + SS + CCS	0.9459	0.9155	0.9796	0.8621	0.9048	0.9453
P + P2	0.9392	0.8959	0.9898	0.7931	0.9048	0.9377
SS + P2	0.9122	0.8742	0.9592	0.7586	0.9048	0.9116
CCS + SS + P2	0.9189	0.8776	0.9694	0.7586	0.9048	0.9177
P + SS + CCS + P2	0.9392	0.8959	0.9898	0.7931	0.9048	0.9377

**Table 4:** The f1 scores obtained for different feature sets and feature set combinations on the performance of different SMOTE-Tomek models in predicting conotoxins that bind to nAChRs.

	SMOTE-Tomek PLR	SMOTE-Tomek PCA PLR	SMOTE-Tomek SVM	SMOTE-Tomek PCA SVM	SMOTE-Tomek PCA RF	SMOTE-Tomek PCA xGB
P	0.9024	0.9078	0.9077	0.8968	0.8241	0.8970
SS	0.8474	0.8743	0.8527	0.8580	0.8860	0.8747
SS + CCS	0.8743	0.8743	0.8690	0.8636	0.8697	0.8751
**P + CCS**	**0.9078**	0.8969	**0.9077**	**0.9131**	0.8796	0.8862
P + SS	0.8807	0.8860	0.9022	0.9022	0.8393	0.8970
P + SS + CCS	0.8915	0.8915	0.8967	0.9076	0.8341	0.8807
P + P2	0.9023	0.8914	0.8965	0.9076	0.8912	0.9133
SS + P2	0.8743	0.8577	0.8524	0.8631	0.8570	0.8858
CCS + SS + P2	0.8468	0.8690	0.8524	0.8687	0.8796	0.8588
P + SS + CCS + P2	0.8859	0.8914	0.8856	0.8856	0.8634	0.8916

**Table 5:** The effect of different feature sets and feature set combinations on the performance of SMOTE-Tomek PCA SVM model in predicting conotoxins that target nAChRs.

	OA	AA	Sn-nAChR Binders	Sn-nAChR Non Binders	f1
P	0.8967	0.8961	0.8902	0.9020	0.8968
SS	0.8587	0.8534	0.8049	0.9020	0.8580
SS + CCS	0.8641	0.8595	0.8171	0.9020	0.8636
**P + CCS**	**0.9130**	**0.9132**	**0.9146**	**0.9118**	**0.9131**
P + SS	0.9022	0.9010	0.8902	0.9118	0.9022
P + SS + CCS	0.9076	0.9059	0.8902	0.9216	0.9076
P + P2	0.8967	0.8937	0.8659	0.9216	0.8965
SS + P2	0.8641	0.8571	0.7927	0.9216	0.8631
CCS + SS + P2	0.8696	0.8632	0.8049	0.9216	0.8687
P + SS + CCS + P2	0.8859	0.8827	0.8537	0.9118	0.8856

## Data Availability

Datasets used in this study are available in File S1.

## Supplementary Materials

The following supporting information can be downloaded at: https://www.mdpi.com/article/10.3390/toxins16110475/s1; Figure S1: Distribution of conotoxin classes; Table S1: Feature sets; Table S2: Sample sizes for alpha, mu, and omega conotoxin classes after being treated with SMOTE-Tomek; File S1: Five datasets used in this study.
