# Supplementary material for: Machine Learning Framework for Conotoxin Class and Molecular Target Prediction
Source: Toxins (Basel). 2024 Nov 3;16(11):475. doi: 10.3390/toxins16110475 (PMC11598409; doi:10.3390/toxins16110475)
Supplement: Supplementary file 1 [file toxins-16-00475-s001.zip › toxins-3181895-supplementary .pdf]

Supplementary Information

Machine Learning Framework for Conotoxin Class and Molecular Target Prediction

Duc P. Truong, Lyman K. Monroe, Robert F. Williams and Hau B. Nguyen

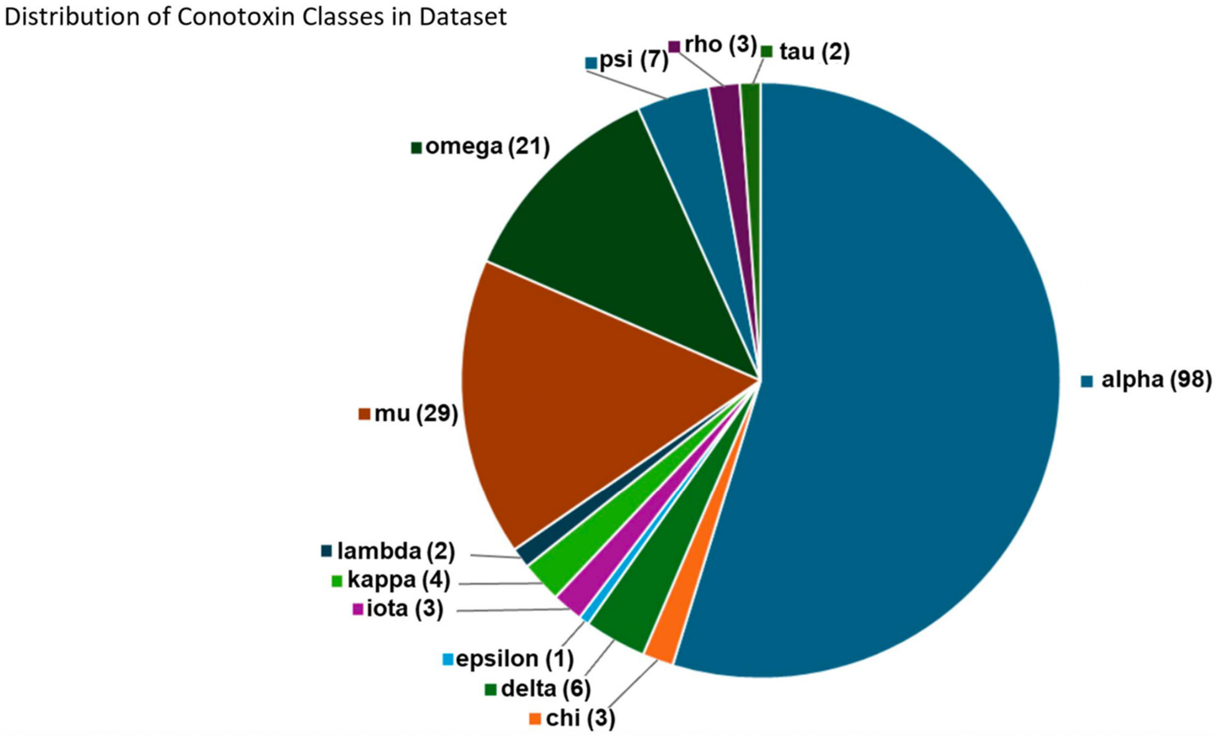

Figure S1. Distribution of conotoxin classes obtained for this study.

Table S1. Feature sets

Feature set	Included features
P	Number of charged residues.<br>Number of aliphatic residues.<br>Number of aromatic residues.<br>Number of polar residues.<br>Number of hydrophobic residues.<br>Number of positively charged residues.<br>Number of negatively charged residues.<br>Number of tiny residues.<br>Number of small residues.<br>Number of large residues.<br>Total charge<br>Mass (kDa)<br>Normalized amino acid counts (number of an amino acid / sequence length)<br>Dipeptide 0<br>Dipeptide 1
P2	Dipeptide 2<br>Number of posttranslational modifications
SS	Number of residues in the DSSP defined structures: G, H, I, T, E, B, S.<br><br>SASA of:<br><br>-Charged residues.<br>-Aliphatic residues.<br>-Aromatic residues.<br>-Polar residues.<br>-Hydrophobic residues.<br>-Positively charged residues.<br>-Negatively charged residues.<br>-Tiny residues.<br>-Small residues.<br>-Large residues.<br>- Posttranslational modifications<br><br>Total SASA.<br><br>Radius of gyration.<br><br>Number of disulfide bonds.<br><br>Disulfide bond geometry (residue number of cystines involved, bond length, bond angles, and dihedral angle around the S-S bond)
CCS	Calculated collisional cross section.

Table S2. Sample sizes for alpha, mu, and omega conotoxin classes after being treated with SMOTE-Tomek.

	P	SS	SS+ CCS	P+CCS	P+ SS	P+SS +CCS	P+ P2	SS+ P2	CCS+SS +P2	P + SS +CCS+P2
Alpha (98)	97	97	97	96	96	96	97	97	96	96
Mu (29)	95	97	96	94	96	96	97	97	96	95
Omega (21)	95	97	96	95	97	97	97	97	97	96

Libraries used in this work:

Perl libraries used for feature extraction: Math

Python libraries used: sys, os, glob, numpy, itertools, pandas, collections, statistics, pickle, csv
